# Supplementary material for: Circulating Monocyte Count as a Surrogate Marker for Ventricular-Arterial Remodeling and Incident Heart Failure with Preserved Ejection Fraction
Source: Diagnostics (Basel). 2020 May 8;10(5):287. doi: 10.3390/diagnostics10050287 (PMC7277943; doi:10.3390/diagnostics10050287)
Supplement: Supplementary file 1 [file diagnostics-10-00287-s001.pdf]

Supplemental Materials

Table 1. Multivariate models examining determinants for CCAD.

	CCAD as Dependent variable					
	**Multivariate Model ¥ (SBP in model)**		**Multivariate Model ¥ (DBP in model)**		**Multivariate Model ¥ (PP in model)**	
	**Coef. (95 % CI)**	***P value***	**Coef. (95 % CI)**	***P value***	**Coef. (95 % CI)**	***P value***
Demographics, n (%)						
**Age, +10 year**	0.22 (0.19, 0.25)	< 0.001	0.26 (0.23, 0.29)	< 0.001	0.22 (0.19, 0.25)	< 0.001
**Sex (men), n %**	0.35 (0.30, 0.41)	< 0.001	0.34 (0.29, 0.40)	< 0.001	0.38 (0.33, 0.44)	< 0.001
**Body mass index, +1 kg/m2**	0.04 (0.03, 0.04)	< 0.001	0.04 (0.03, 0.05)	< 0.001	0.04 (0.03, 0.05)	< 0.001
**SBP, +10 mmHg**	0.09 (0.08, 0.11)	< 0.001	NA	NA	NA	NA
**DBP, +10 mmHg**	NA	NA	0.10 (0.07, 0.13)	< 0.001	NA	NA
**Pulse pressure, +10 mmHg**	NA	NA	NA	NA	0.11 (0.08, 0.13)	< 0.001
**Heart rate, +10 bpm**	—	—	—	—	—	—
Past Medical History, n (%)						
**Hypertension**	0.13 (0.05, 0.21)	0.004	0.16 (0.08, 0.24)	< 0.001	0.16 (0.08, 0.24)	< 0.001
**Diabetes mellitus**	—	—	—	—	—	—
**CVD**	—	—	—	—	—	—
**Hyperlipidemia**	—	—	—	—	—	—
**Gout**	0.13 (0.02, 0.23)	0.017	0.13 (0.03, 0.24)	0.013	0.13 (0.02, 0.23)	0.016

Laboratory Data						
**Glucose, +10 mg/dL**	0.01 (0.003, 0.03)	0.015	0.02 (0.004, 0.03)	0.007	0.01 (0.003, 0.03)	0.013
**Cholesterol, +10 mg/dL**	—	—	—	—	—	—
**HDL-c**	—	—	—	—	—	—
**eGFR, +10 mL/min/1.73 m^2**	—	—	0.01 (-0.002, 0.03)	0.09	—	—
Life Style Modification						
**Active smoking**	0.15 (0.05, 0.24)	0.002	0.15 (0.05, 0.24)	0.002	0.14 (0.04, 0.23)	0.005
**Regular exercise**	-0.08 (-0.16, 0.01)	0.095	—	—	-0.08 (-0.17, 0.006)	0.067

Abbreviations as Table 1.

NA = not available in model, ¥ systolic blood pressure (SBP), diastolic blood pressure (DBP), and pulse pressure (PP) were separately entered into multivariate models due to collinearity. Other abbreviations see Table 1.
